# The sequence spectrum of frameshift reversions obtained with a novel adaptive mutation assay in *Saccharomyces cerevisiae*

**DOI:** 10.1016/j.dib.2016.11.061

**Published:** 2016-11-22

**Authors:** Erich Heidenreich, Ferdinand Steinboeck

**Affiliations:** Institute of Cancer Research, Department of Medicine I, Medical University of Vienna, Borschkegasse 8a, A-1090 Vienna, Austria

**Keywords:** Adaptive mutation, Selection-induced, Stress-induced, Spontaneous mutagenesis

## Abstract

Research on the mechanisms of adaptive mutagenesis in resting, *i.e.* non-replicating cells relies on appropriate mutation assays. Here we provide a novel procedure for the detection of frameshift-reverting mutations in yeast. Proliferation of non-reverted cells in this assay is suppressed by the lack of a fermentable carbon source. The test allele was constructed in a way that the reversions mimic microsatellite instability, a condition often found in cancer cells. We show the cell numbers during these starvation conditions and provide a DNA sequence spectrum of a representative set of revertants. The data in this article support the publication "Glucose starvation as a selective tool for the study of adaptive mutations in *Saccharomyces cerevisiae*" (Heidenreich and Steinboeck, 2016) [1].

**Specifications Table**

**Table:** 

Subject area	*Biology*
More specific subject area	*Microbiology, Mutation Research*
Type of data	*Table, Figures*
How data was acquired	*Cell counts by microscopy; DNA sequencing*
Data format	*Raw, analyzed*
Experimental factors	*Random choice of revertants from different time points (for sequencing)*
Experimental features	*A novel mutation assay under glucose-limited conditions was performed. Cell numbers during glucose starvation were assessed. The test allele of a representative number of revertants was sequenced.*
Data source location	*Vienna, Austria*
Data accessibility	*Data are presented in this article*

**Value of the data**

• We share the protocol for a new adaptive mutation assay in *Saccharomyces cerevisiae* that will be the new standard assay in our lab. We want to encourage others to use it as well.
• As a part of quality assurance, we demonstrate the lack of proliferation during protracted starvation.
• We provide the first sequence spectrum of reversions of the novel *fbp1MS2* test allele to serve as a benchmark for future comparisons.

## 1 Data

**Fig. 1:**
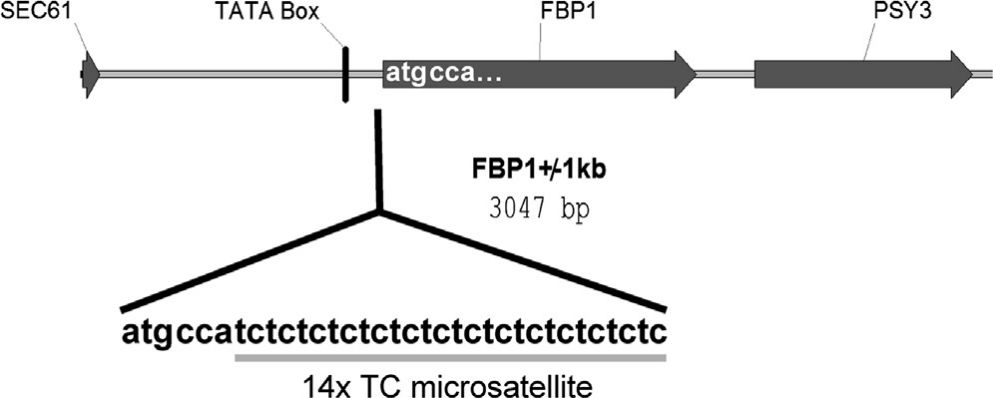
Layout of the *fbp1MS2* allele. A custom-designed oligonucleotide containing a 14-fold repeat of a TC dinucleotide was integrated in front of the native *FBP1* start codon.

**Fig. 2:**
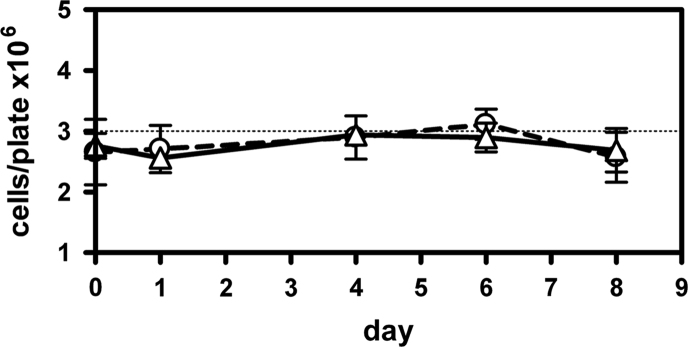
Time-course of cell numbers after transfer of cells from liquid preculture to glucose-less lactate medium plates at a target density of 3×10^6^ cells per plate (shown as a dotted line). The Fbp1 knockout strain EHDF1 (circles, dashed line, *n*=3) and the *fbp1MS2* strain EHFMS2 (triangles, solid line, *n*=4) both experience a strict cell cycle arrest on these starvation plates.

**Fig. 3:**
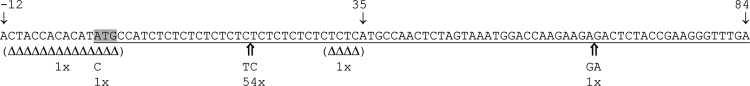
Mutational spectrum of 58 revertants of the *fbp1MS2* allele. Numbering above the sequence is in relation to the translation start (start codon is shaded), deleted stretches are indicated by (Δ), a gain of one TC repeat in the engineered TC microsatellite and one GA repeat in an innate GA repeat is indicated by an arrow (⇑). The reversion window, where potential reversions have to be located is underlined.

DNA replication is the major source of mutations in proliferating cells. However, mutations also arise in resting cells. The mechanisms responsible for the latter are less well understood than replication-dependent mutagenesis. The most interesting subset of mutations in resting cells are such that appear to be adaptive, *i.e.* that provide a selective advantage to the mutants by enabling a resumption of proliferation. For the study of such adaptive mutations, a combination of a useful test allele and appropriate cell cycle-arresting but non-lethal conditions is necessary [2]. In this article, we present data on the implementation of a novel adaptive mutation assay (Fig. 1 and Table 1) as a tool to generate adaptive revertants for further analysis. We monitored the quality of the induced cell cycle arrest (Fig. 2) and provide a sequence analysis of representative sets of revertants (Fig. 3 and Table 2).

## 2 Experimental design, materials and methods

### 2.1 Components of the mutation assay

The yeast strain YFMS2 carries a custom-designed microsatellite sequence as a target for spontaneous reversions (Fig. 1). Details of the construction are presented in [1]. Translation of the resulting *fbp1MS2* allele is truncated by a frameshift.

Starvation for glucose (on lactate medium) drives YFMS2 cells into a cell cycle arrest that permits the selection for revertants able to resume proliferation by a mutational reversion of the *fbp1MS2* frameshift. The media used (YPD and SC) have been described by Sherman [3]. SC/Lactate is SC medium with 3% DL-lactate instead of 2% glucose. The detailed experimental procedure of the new adaptive mutation assay is listed in Table 1.

### 2.2 Data on the strictness of starvation-induced cell cycle arrest

Any variation in cell numbers during starvation for glucose was monitored by counting the cells washed from representative parallel plates treated identical to the mutation assay. Data obtained with the assay strain YFMS2 were compared to data obtained with a *FBP1* knockout strain named EHDF1 (Fig. 2). A leaky cell cycle arrest would be evident by an increase in cell numbers.

### 2.3 DNA sequence data

Revertant colonies obtained in *fbp1MS2* mutation assays performed according to Table 1 were isolated and their genomic DNA prepared. A fragment containing the *FBP1* allele was amplified by PCR and after purification sequenced by a contractor (VBC genomics, Vienna, Austria). The clones have been randomly chosen among those appearing either on day 4 (*i.e.* replication-dependent revertants carried over from preculture), day 8 (early adaptive mutants) or days 11–13 (late adaptive revertants). The type and the location of the genetic alterations of 58 revertants is shown in Fig. 3. Table 2 lists the distribution of mutation types among the three temporal groups. With this number of clones, there is neither a significant difference in the mutational spectrum between the three temporal groups nor between the replication-dependent revertants and the total of adaptive revertants (*p*>0.05 with an algorithm by Adams and Skopek [4]).

## Figures and Tables

**Table 1:** Protocol for the *fbp1MS2* adaptive mutation assay.

DAY -3 (Monday recommended):
• Plate 40 colony-forming units (cfu´s) of the test strain on three YPD plates each. Incubate at 30 °C
DAY -1
• Prepare about 50 SC/Lactate plates. 32 plates of similar thickness are needed (mean weight +/−5%, to provide a similar amount of nutrients on each plate). • Inoculate 7 (+1 backup) tubes of 4 ml YPD each with an equally sized individual colony of the test strain (cultures A-H) • Incubate o/n shaking at 30 °C
DAY 0
• Transfer 1 ml of each preculture to a microcentrifuge tube • Harvest cells by centrifugation at 5000 rpm for 3 min • Discard supernatant • Wash each cell pellet with 1 ml sterile water • Centrifuge with same settings and discard supernatant • Discard the tube of the backup culture H if all the others are okay • Resuspend each cell pellet of cultures A-G in 1 ml sterile water • Prepare two serial 1/10 dilutions of cultures A, B and C for counting and determine the cell densities of these three o/n-cultures (hemocytometer count of 1/100 dilution) • Use the mean of the cell counts of cultures A, B and C (previous step) to calculate the volume (of the undiluted cell suspensions) containing 2×107 cells • Remove this volume from the undiluted A- to G-tubes, transfer it to fresh tubes and fill up to 1 ml with sterile water to result in a density of 2×107 cells/ml • Place aliquots of 150 μl on a series of 6 (for A & B) and 4 (C to G) SC/Lactate plates, respectively. This results in 3×106 cells per plate • Use one Drigalski spatula to evenly spread the cells on all six and all four plates, respectively (labelled 1–6 and 1–4 respectively) • Incubate plates at 30 °C in normal incubator • Determination of cell numbers and viability: After soaking in, rinse cells off the plates A5 and B5 (in parallel) with a total of 4 ml sterile water (add 2 ml sterile water, release cells with a Drigalski spatula, transfer suspension to a 15 ml tube, repeat twice with 1 ml water). Measure the volume of the collected liquid with the help of a 5 ml glass pipet. Directly determine cell density with a hemocytometer. Prepare appropriate dilutions and plate about 100 cfu´s on each of three YPD plates for survival determination. Calculate the mean of the two counts
DAY 1
• Determination of cell numbers and viability: Rinse cells off the plates A6 and B6 (in parallel) with a total of 4 ml sterile water (add 2 ml sterile water, release cells with a Drigalski spatula, transfer suspension to a 15 ml tube, repeat twice with 1 ml water). Measure the volume of the collected liquid with the help of a 5 ml glass pipet. Directly determine cell density with a hemocytometer. Prepare appropriate dilutions and plate about 100 cfu´s on each of three YPD plates for survival determination. Calculate the mean of the two counts • Transfer all plates to a humidified (e.g. cell culture) incubator (30 °C)
DAYS 4 to 15
• Count and mark newly emerging colonies (larger than 0.5 mm diameter) on all plates

**Table 2:** Incidence of the mutation types shown in Fig. 3 grouped by their origin (day of colony appearance during mutation assay indicated).

**Mutation type**	**Incidence among replication-dependent revertants (day 4)**	**Incidence among early adaptive revertants (day 8)**	**Incidence among late adaptive revertants (days 11–13)**
Gain of one TC repeat	20	16	18
Gain of one GA repeat		1	
Loss of two TC repeats		1	
Deletion of 14 base pairs incl. start codon			1
Base substitution A>C in start codon			1
total	20	18	20
