# Supplementary material for: The sequence spectrum of frameshift reversions obtained with a novel adaptive mutation assay in Saccharomyces cerevisiae
Source: Data Brief. 2016 Nov 22;9:1113–7. doi: 10.1016/j.dib.2016.11.061 (PMC5128011; doi:10.1016/j.dib.2016.11.061)
Supplement: Supplementary file 1 — Supplementary material [file mmc1.doc]

Although announced in the online form ("Conflict of Interest is mandatory for revision, so while submitting please submit the file by selectin the description from the drop down. Please click here to download the Conflict of Interest form."), there is no link to a conflict of interest form. Unfortunately, I couldn´t find it elsewhere either.

Anyway, I declare that there is no conflict of interest.

If, you need a special form, please send it to me.

best regards,

Dr. Erich Heidenreich

email: erich.heidenreich@meduniwien.ac.at
